# Ultrafast kinetics of linkage isomerism in Na_2_[Fe(CN)_5_NO] aqueous solution revealed by time-resolved photoelectron spectroscopy

**DOI:** 10.1063/1.4990567

**Published:** 2017-06-28

**Authors:** Azhr A. Raheem, Martin Wilke, Mario Borgwardt, Nicholas Engel, Sergey I. Bokarev, Gilbert Grell, Saadullah G. Aziz, Oliver Kühn, Igor Yu. Kiyan, Christoph Merschjann, Emad F. Aziz

**Affiliations:** 1 Department of Physics, Freie Universität Berlin, Arnimallee 14, 14195 Berlin, Germany; 2 Joint Laboratory for Ultrafast Dynamics in Solutions and at Interfaces (JULiq), Institute of Methods for Material Development, Helmholtz-Zentrum Berlin, Albert-Einstein-Strasse 15, D-12489 Berlin, Germany; 3 Department of Physics, College of Science, University of Karbala, 56001 Karbala, Iraq; 4 Institut für Physik, Universität Rostock, Albert-Einstein-Str. 23-24, D-18059 Rostock, Germany; 5 Chemistry Department, Faculty of Science, King Abdulaziz University, 21589 Jeddah, Saudi Arabia; 6 School of Chemistry, Monash University, 19 Rainforest Walk, Melbourne, Victoria 3800, Australia

## Abstract

The kinetics of ultrafast photoinduced structural changes in linkage isomers is investigated using Na_2_[Fe(CN)_5_NO] as a model complex. The buildup of the metastable side-on configuration of the NO ligand, as well as the electronic energy levels of ground, excited, and metastable states, has been revealed by means of time-resolved extreme UV (XUV) photoelectron spectroscopy in aqueous solution, aided by theoretical calculations. Evidence of a short-lived intermediate state in the isomerization process and its nature are discussed, finding that the complete isomerization process occurs in less than 240 fs after photoexcitation.

## I. INTRODUCTION

Light-induced metal-to-ligand charge transfer (MLCT) plays an important role in the photophysics and photochemistry of organometallic coordination compounds,^1–4^ including such reactions as photochemical substitution, isomerization, and radical formation.^5–7^ MLCT transitions thus reflect an efficient way of light harvesting in specific wavelength ranges.^8,9^ Thermodynamically, MLCT excited states are unstable, decaying via either light emission, or the subsequent formation of metastable (MS) states with lifetimes typically larger than 1 ns.^8,9^ The latter generally possess a different electronic and geometrical structure compared to the ground state (GS),^10,11^ thus facilitating the conversion of light into chemical, electrical, or potential energy.^8^ A special class of MS states is found in the so-called linkage isomers.^12^ These compounds exhibit geometrical rearrangements of one or more ligands with respect to the central metal atom.^12^ Since the rearrangement mechanisms initially require a formal oxidation of the metal center, MLCT forms the basis of phototriggered linkage isomerization.

**FIG. 1.**
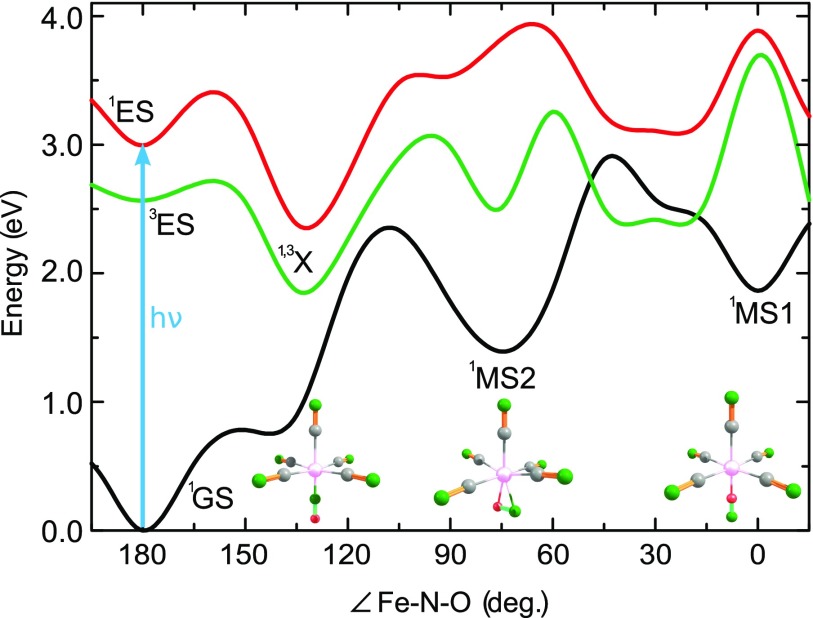
Calculated minimum energy path on the ground state potential energy surface along the coordinate of linkage isomerization (relaxed potential for the fixed Fe–N–O valence angle), shown as the black curve. Also shown are cuts of the potential energy surfaces of the lowest excited singlet (red) and triplet (green) electronic states along this ground state minimum energy path. The geometric configurations of the GS, MS1, and MS2 minima are shown as insets. For the labeling, see text.

A large family of complexes showing linkage isomerism are nitrosyl compounds with the general composition [ML_*x*_(NO)]^*n*^, where M represents a transition metal (e.g., Fe, Ni, Ru, and Os) and L denotes a ligand such as F^−^, Cl^−^, CN^−^, NH_3_, and ${\text{NO}}_{2}^{-}$.^9,13,14^ Among these complexes, Na_2_[Fe(CN)_5_NO] (sodium nitroprusside, SNP) is a prototype system.^15^ Besides its application as a blood-pressure-regulative agent,^16^ this compound has received much attention in the last few decades due to the ease of investigating charge-transfer processes and isomerization reactions.^17–21^ Potential applications include light-induced N-O release for photodynamic therapy,^22^ optical switching and dynamic holography,^23,24^ and photoinduced chemical reactions.^14^ Being initially discovered by Mössbauer spectroscopy in 1977,^18^ the photophysics of SNP has been extensively studied in crystals,^25–29^ and more recently also in solutions, where the [Fe(CN)_5_NO]^2−^ ion is spatially separated from its counter-ion.^17,30–32^ These studies revealed that the final product of the isomerization reaction depends on the applied photon energy. This can be understood by considering cuts of the potential energy surfaces along the coordinate of linkage isomerization (corresponding to geometry-optimized configurations in the electronic ground state for the given Fe–N–O valence angle), shown in Fig. 1 for the ground state (black curve), as well as for the lowest excited singlet (red) and triplet (green) electronic states along this ground state minimum energy path. Under irradiation with blue-green light (450–560 nm), SNP is promoted from its singlet ground state (GS) with ∠Fe−N−O=180° to the MLCT excited state (ES), corresponding to the transition from the highest occupied molecular orbital Fe(3d_*xy*_) to the lowest unoccupied orbital $\pi_{\text{NO}}^{*}$.^33^ This excitation transition triggers the geometrical reorganization of the Fe–NO bond towards an almost orthogonal (∠Fe−N−O≈77°) side-on metastable configuration of NO, named MS2.^25,28,29^ Using light of shorter wavelength (<450 nm) results in the population of a higher-lying excited state and subsequently leads to the formation of the isonitrosyl (Fe–ON) metastable configuration (MS1),^25,28,29^ corresponding to a higher-lying local minimum of the ground state potential energy surface at 0°. Note that this latter configuration is not the subject of the current work.

Extended irradiation can also lead to re-excitation from MS1 or MS2 into higher-lying excited states, from where relaxation into the other electronic ground or metastable states (GS, MS1, and MS2) is possible.^34,35^ In general, the spontaneous relaxation from MS states towards GS proceeds non-radiatively, and is thermally activated. Utilizing nanosecond transient absorption (TA) spectroscopy at ambient temperature, Schaniel *et al.* have determined the corresponding lifetime of MS2 to be 300 ns in single crystals and 110 ns in aqueous solution.^17^ Thus, the population density of MS1/MS2 also depends on the intensity and duration of the applied irradiation, as well as on temperature.^34,35^

More recent femtosecond TA studies in the UV/Vis/NIR range addressed the initial relaxation processes from ES to the MS states. They revealed that the relaxation from ES to MS2 is monoexponential with a time constant of ≤300 fs in single crystals; the concurrent direct back transfer from ES to GS was found to proceed in the same time range, and the over-all population-transfer efficiency from GS to MS2 was about 10%.^24,36^ Complementary picosecond IR TA experiments by Lynch *et al.* in methanol solutions have confirmed the previously reported values for the lifetimes of MS1 and MS2 (i.e., the relaxations from MS towards GS states).^32^ The IR TA study further revealed evidence for an ultrafast transition (≤25 fs) from ES to an intermediate state X, from which MS2 was assumed to be populated with a time constant of 300 fs.^32^ However, the limited time resolution of the IR TA experiments did not allow for decisive studies of these early-time dynamics of photoexcited SNP in detail. In particular, despite extensive investigations, the nature of the initial processes including the lifetime of the ES state, the pathways to the metastable states, and the absolute electron binding energies of the involved states remain unclear until today.

In the present work, we apply femtosecond transient photoelectron spectroscopy (PES), aided by theoretical calculations, to investigate the excitation to the MLCT state and the subsequent early-time dynamics of the NO ligand of SNP in aqueous solution. After excitation with light at 500 nm (2.48 eV photon energy), the electron photoemission yield upon probing with extreme ultraviolet (XUV) photons provides information about the lifetime of the ES (MLCT) state, as well as the build-up dynamics of the MS2 state, and the electronic binding energies of GS, ES, X, and MS2. The time resolution of <70 fs achieved in the present experiment allows us to track the electron dynamics on a time scale shorter than those reported previously. Our results refine the kinetic models featured in the literature.^24,32,36^ The experimental findings are supported by density functional theory (DFT) calculations.

## II. EXPERIMENTAL SETUP AND MATERIAL

### A. Transient PES setup

Visible pump and XUV probe pulses are generated by using a commercial Ti:sapphire laser system, delivering pulses of 25 fs duration at 800 nm central wavelength and 2.5 mJ pulse energy with a repetition rate of 5 kHz. The laser output is split by a beam splitter, so that approx. 1 mJ of the pulse energy is applied to pump an optical parametric amplifier (OPA), generating visible pulses at 500 nm wavelength (2.48 eV photon energy). Another split beam is used to pump a high-harmonic-generation (HHG) setup to produce XUV probe pulses. The beam of the 21st harmonic (photon energy of 32.55 eV) is selected by using a reflection off-center zone plate,^37,38^ and refocused by a toroidal mirror into the experimental chamber. This HHG setup is described in more detail by Metje *et al.*^39^ The volatile sample solution is introduced into the interaction region using a micro-jet technique.^40,41^ Passing through a quartz nozzle of approx. 24 *μ*m diameter at a flow rate of 0.4 ml/min, the liquid jet remains laminar over a distance of approx. 2–3 mm from the nozzle tip. After the laminar region, the liquid flow breaks up into droplets, which are collected in a LN_2_-cooled liquid trap. This ensures the working pressure to be kept below 2 × 10^−5^ mbar in the interaction chamber, required for electron detection. In the actual experiment, the spot size of the HHG probe beam at the liquid jet was 100 *μ*m, delivering approx. 10^6^ photons per pulse in the interaction region, as measured by a photo-diode. The spot size of the 500 nm pump beam was 200 *μ*m, with a maximum pulse energy of 1.5 *μ*J. Its pulse duration (FWHM) of 55 fs was determined using an optical autocorrelator. Pump and probe beam polarization was mutually parallel, under an incidence angle of 1° between the two beams. The pump-probe delay time was adjusted by an optical delay line for the pump beam, allowing for a resolution of 6.6 fs and a maximum delay of 2 ns. The time-response of the apparatus is defined by the width of the cross-correlation (CC) trace of (63.8 ± 0.7) fs (FWHM), obtained directly from time-resolved PES measurements in SNP solutions. The liquid jet is centered at a distance of approx. 1 mm in front of the skimmer orifice (*d* ≈ 400 *μ*m) of a commercial time-of-flight (TOF) electron spectrometer (SPECS, THEMIS 600), which consists of a drift tube with a set of electrostatic lenses and a microchannel plate detector (MCP) at the end of the tube. The lens system focuses the photoelectrons that pass through the tube to the detector, allowing the operation of the TOF in either drift mode or wide-angle mode. The former reveals higher energy resolution, while the latter allows for faster data acquisition. It is only in wide-angle mode that transient spectra can be recorded with reasonable acquisition time. The lens electrodes and detector were wrapped with two magnetically isolating layers (*μ* metal shielding) to decrease the effect of external magnetic fields (including the earth's magnetic field) down to an uncritical level. To avoid a saturation of the electron detector, a deceleration voltage to a grid positioned in front of the detector has been applied.

### B. Material

Crystalline SNP (Sigma-Aldrich Co., purity >99.9%) was dissolved in distilled milli-Q water to prepare an aqueous solution of 500 mM. Sodium chloride with concentration 20 mM was added to the SNP solution to increase the conductivity and to decrease the streaming potential caused by friction between the sample and nozzle during flowing.^42^ The absorption spectrum of the sample solution was obtained by using a UV/Vis spectrophotometer (Implen, Nano). All measurements were performed at ambient temperature. The pump photon energy (2.48 eV) corresponds to the singlet-singlet MLCT transition of ${2b}_{2}({3d}_{xy})\to 13e(\pi_{\text{NO}}^{*})$ character, as depicted in the absorbance spectrum (see Fig. 6 in the Appendix).^33^

## III. COMPUTATIONAL DETAILS

### A. Electronic structure

All calculations were performed at the DFT level employing the range-separated LC-BLYP functional,^43^ where the range separation parameter was optimized according to a ΔSCF procedure as described in detail in Refs. 44 and 45. For [Fe(CN)_5_NO]^2−^, the optimal value of this parameter was found to be 0.21 bohr^−1^. Utilization of the optimally tuned range-separation functional allows us to improve the reliability for the optical and especially photoelectron spectra due to the mitigation of the electron self-interaction error.^46^ All DFT calculations have been performed using the GAUSSIAN 09 package^47^ with the cc-pVTZ basis set.^48,49^ The solvent environment was accounted by the polarizable continuum model^50^ which is essential to stabilize the complex. The GS, MS1, and MS2 minima of the singlet ground state potential energy surface have been obtained as well as the adiabatic minimum energy path along the NO rotation coordinate. Five excited singlet states have been calculated along this ground state path at the level of time-dependent DFT (TDDFT) as well as one triplet state at the level of unrestricted DFT (UDFT). Their cuts along the coordinate of linkage isomerization (relaxed potential for the given Fe–N–O valence angle) are presented in Fig. 1. One should point out again that the electronic ground state exhibits three minima, corresponding to GS, MS1, and MS2, respectively. The respective geometrical configurations of the molecule are depicted in the insets of Fig. 1. Note that there are two common definitions for the isomerization-angle coordinate: Fe–N–O valence angle^51^ and center-of-mass angle.^52^ We have chosen the former, resulting in apparent differences to other literature values, most prominently the angular positions of GS, MS1, and MS2, which in our case appear at 180°, 0°, and 77°, respectively.

### B. Photoelectron spectra

To interpret the experimental data, the photoelectron probe spectrum has been computed for the ground and lowest excited singlet and triplet initial states at the GS and MS2 as well as at a slightly skewed (∠Fe−N−O=135°, denoted as X) geometry. The 150 final doublet states of the ionized system were computed with the Tamm-Dankoff approximation to TDDFT with cc-pVTZ basis and PCM as described above. The photoionization cross sections were calculated employing the Dyson orbital approach as described in Ref. 53. The numerical evaluation of the bound-continuum transition matrix elements has been performed by the ezDyson v3.0 program.^54^ The final state of the photoelectron is therein represented by a plane wave expanded in terms of spherical waves up to angular momentum truncated at *l*_max_ = 7. The numerical integration was carried out on a three-dimensional uniform grid in a box with a side length of 12 Å and 360 grid points per dimension. Since the ionization intensities are proportional to the respective squared Dyson orbital norms, they were calculated only if the respective values were larger than 10^−4^.

## IV. RESULTS AND DISCUSSION

**FIG. 2.**
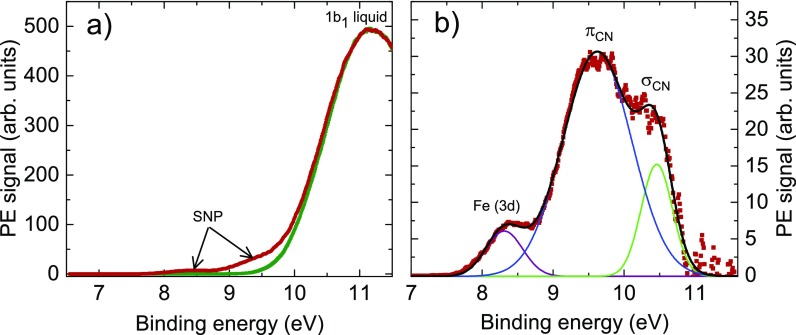
(a) Steady-state XUV photoemission spectra, recorded in drift mode, of pure water (green) and SNP dissolved in water (red). (b) Difference spectrum showing the SNP spectral components in more detail. The data have been fitted with a superposition of three Gaussian profiles, depicted as solid curves. The individual bands at 8.25 eV, 9.62 eV, and 10.45 eV are attributed to ionizations from Fe(3d), mainly *π*_CN_ orbitals, and predominantly *σ*_CN_ ligand orbitals, respectively.

Figure 2(a) shows the steady-state XUV photoemission spectra of the pure solvent (green) and SNP aqueous solution (red), recorded in the drift operational mode of the TOF spectrometer. One can clearly observe the contributions of SNP in the region below 11 eV binding energy. These SNP-related signals are shown in more detail in Fig. 2(b), obtained after subtraction of the solvent signal as a background. Three spectral bands, centered at binding energies of 8.25 eV, 9.62 eV, and 10.45 eV, are distinguishable. These energy positions are obtained by fitting the background-subtracted signals with a superposition of three Gaussian profiles [see Eq. (A5)]. According to our TDDFT calculations, as well as previous assignments of XPS/UPS spectra by Gädeke *et al.*,^55^ the band with the lowest binding energy of approximately 8–9 eV corresponds to ionization from the Fe(3d) orbitals, whereas the other bands lying above 9 eV have dominant contributions from CN^−^ ligands. In particular, the band at approximately 9.6 eV corresponds predominantly to ionization from *π*_CN_ orbitals, those at ≥10 eV stem from *σ*_CN_ orbitals. Interestingly, photoionization from the NO^+^ ligand does not contribute to the ionization cross section in the shown energy range.

**FIG. 3.**
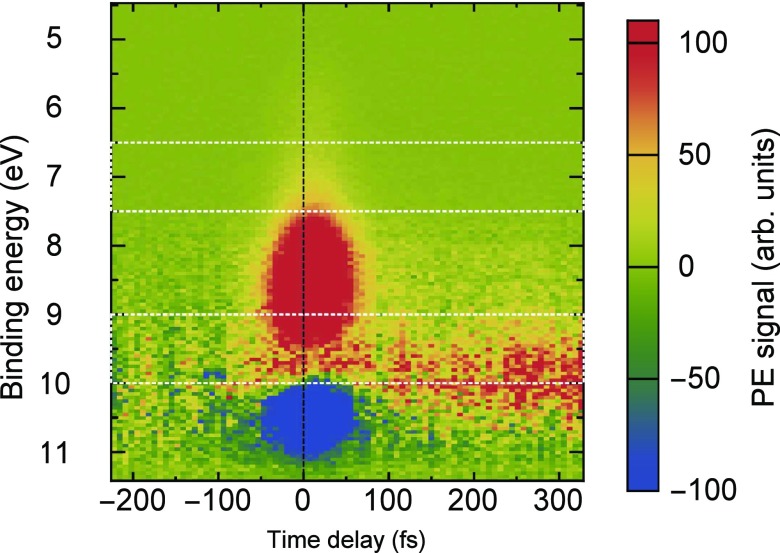
Transient PE signal of SNP aqueous solution dependent on electron binding energy and pump-probe time delay. For better visibility, the averaged spectra recorded at negative delays have been subtracted as a background. The marked areas denote the energy integration regions shown as cuts in Fig. 4.

The results of the time-resolved PES measurements (recorded in wide-angle mode) are presented in Fig. 3, which shows the transient photoemission signal dependent on electron binding energy and pump-probe time delay. Negative time delays imply that the XUV probe pulse arrives first to the interaction region. For better visibility of the transient signal at positive time delays, the averaged spectra recorded at negative delays have been subtracted as a background. The background consists of a superposition of the photoemission spectra of GS and water (see Fig. 2). This leads to a negative signal in the vicinity of zero time delay and at binding energies between 10 and 11 eV, where the photoemission yields of the 1b_1_ orbital of liquid water and of GS are decreased due to the cross-correlation (CC) between pump and probe pulses.^56^ The CC also gives rise to a prominent positive signal between 7.5 and 9.5 eV, which is assigned to the respective photoemission bands of water and SNP, resulting from absorption of one XUV photon and one visible pump photon. Regarding this first-order sideband of the laser-assisted XUV ionization, its spectral yield can be simply represented by the steady-state XUV photoemission spectrum, shifted on the energy axis by the pump photon energy (2.48 eV) towards lower binding energies. A similar—but more sophisticated—consideration was applied previously in Ref. 56. These CC signals can be utilized to determine the time response of the setup, as well as to pin down the exact position of the origin of the delay-time axis, as has been shown by Hertel *et al.*^57^ From the Gaussian fit of the integrated PES signal between 5.55 and 6.55 eV, where we do not observe excited-state dynamics, we determine a time response of (63.8 ± 0.7) fs (FWHM), and assign the zero time delay to the maximum position of the CC trace (see the Appendix, Fig. 7, for details). This pinpointed determination of time zero facilitates the identification of ultrafast resonant contributions to the kinetics with time constants even below the time response of the system: such short-lived signals lead to an apparent shift and asymmetric broadening of the CC-dominated kinetics.^57^

Besides the strong CC signals, there is clear evidence for enhanced photoemission at positive delays, especially between 9.5 and 10.5 eV binding energy. These features emerge during the first 300 fs, and persist beyond the maximum time delay of the current investigation (+330 fs). Therefore, the long-lived features are assigned to MS2, in accordance with previous reports.^24,32,36^ We note that the spectral position and shape of the emerging MS2 PES signal appear similar to that of GS; this finding is also supported by the theoretically calculated PES spectra of GS and MS2 (see Fig. 5 and the discussion below). According to these results of calculations for the singlet and triplet excited states, additional features should appear at binding energies below 8 eV, which are due to electron removal from the $\pi_{\text{NO}}^{*}$ orbital.

**FIG. 4.**
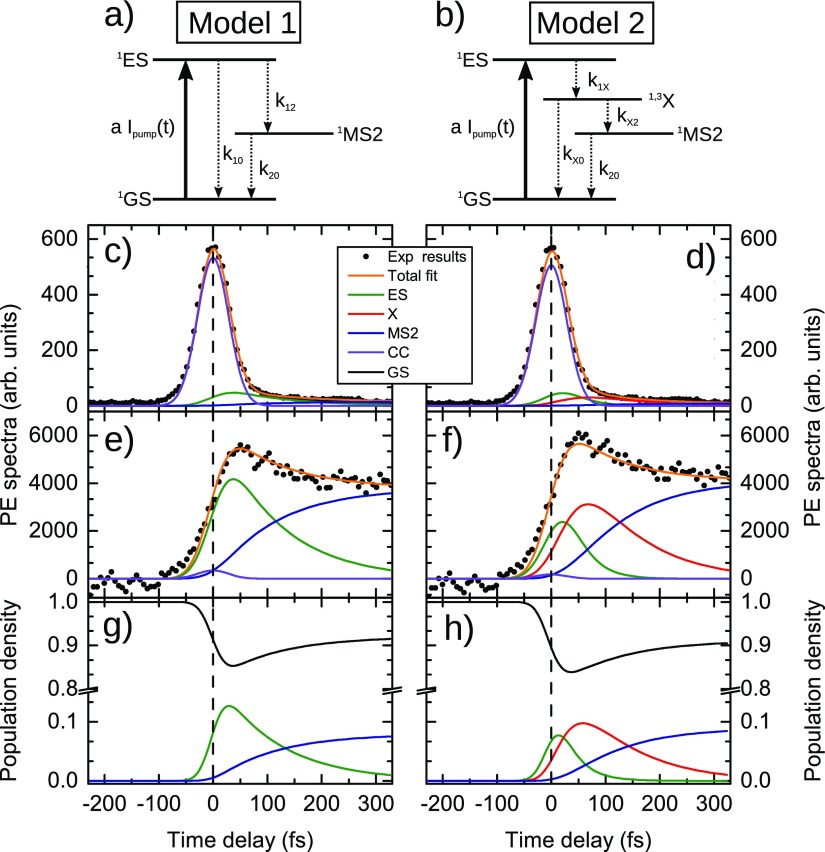
Comparison of global-fit results for models 1 (left column) and model 2 (right column). (a) and (b) Schematic depiction of the respective excitation-relaxation processes. The relevant rate parameters used in Eqs. (A2) and (A3) are given. (c) and (d) Energy-integrated kinetic traces, global fit result, and its decomposition into specific state contributions in the region 6.5–7.5 eV. (e) and (f) Same as (c) and (d), but for the region 9.0–10.0 eV. (g) and (h) Transient population densities obtained from the fits for the GS and excited states of SNP.

Next, we analyze the transient spectra to reveal the ultrafast kinetics during the emergence of the MS2 conformer. Initial photoexcitation (vertical arrow in Fig. 1) populates the lowest singlet state ^1^ES. As one can see from the potential curves in Fig. 1, the skewed (≈135°) geometric configuration of the excited singlet state, denoted as ^1^X, corresponds to the energy minimum lying close to the position of the ground state barrier between the ^1^GS and ^1^MS2 configurations (see also Refs. 51 and 52) and should be considered on a way to ^1^MS2. In addition, the involvement of the triplet ^3^ES state might be possible during the early photodynamics as can be seen from Fig. 1. Based on these considerations as well as kinetic schemes utilized earlier in the literature, we apply two different kinetic models in our analysis, which are depicted schematically in Fig. 4. In model 1, originally used in Refs. 24 and 36, a direct population of MS2 and GS from the MLCT state (ES) is assumed. Model 2 incorporates an additional intermediate state X, which, according to Lynch *et al.* is populated from ES on an ultrafast time scale,^32^ and from where the subsequent parallel relaxation to either MS2 or GS takes place. The nature of this X state in the model 2 will be discussed below. The population dynamics of the short-lived X state should lead to characteristic shifts in the transient photoemission signal on a few-10-fs time scale.^57^ Applying the respective rate equations in a global-fit-analysis reveals kinetic parameters (i.e., rate constants), as well as PES spectra for the involved states. Details about the fit routine are given in the Appendix.

The respective best fits and corresponding residuals for model 1 and model 2 are shown in Fig. 8 (see the Appendix). Note that in analogy to the measured data, the photoemission spectra of GS and water, as obtained from the respective fit, have been subtracted from the calculated transient spectra. The kinetic fit parameters are presented in Table I, whereas the parameters for the Gaussian deconvolution of the fitted XUV photoemission spectra [see Eq. (A5)] are given in Table II in the Appendix. One can see from Fig. 8 that there is no clear superiority of either model in the description of our experimental data. Also, both models are in over-all good agreement concerning the rate parameters. To compare Models 1 and 2 in more detail, Figs. 4(c)–4(f) show the decomposition of the transient signal into individual contributions from the involved ground and excited states, as well as the CC signal. This decomposition is presented for two energy ranges, 6.5–7.5 eV [(c) and (d)] and 9.0–10.0 eV [(e) and (f)], where the respective photoemission yields of ^1^ES and ^1^MS2 are maximal, according to the global fit results. Again, these cuts show that both models provide fit results of the same quality. It is interesting to note that the ionization yield of ^1^ES in model 2 has a maximum around +20 fs. Such a short-time maximum was expected (see above). The decomposed amplitude of the ^1^ES contribution is significant, which supports the presence of an intermediate state X, as suggested in Ref. 32 and by our calculations (cf. Fig. 1).

**TABLE I.** Kinetic fit parameters for model 1 and model 2, respectively.

Parameter	Model 1	Model 2	Unit
*A*	4.00 ± 0.30	4.00 ± 0.57	ps^−1^
*k*_10_	5.00 ± 0.53		ps^−1^
*k*_12_	3.95 ± 0.29		ps^−1^
*k*_1X_		30.0 ± 1.4	ps^−1^
*k*_X0_		4.987 ± 0.065	ps^−1^
*k*_X2_		4.99 ± 0.92	ps^−1^
*k*_20_	9.09 × 10^−6^	9.09 × 10^−6^	ps^−1^
*σ*_pump_	19.60 ± 0.89	19.60 ± 0.82	fs
*σ*_probe_	20.5 ± 1.2	20.5 ± 1.8	fs

**TABLE II.** Fit parameters of Gaussian peaks used to describe the amplitude spectra for models 1 and 2, respectively. Position values denote binding energy.

State	Position/eV	FWHM/meV	Amplitude/cts	Position/eV	FWHM/meV	Amplitude/cts
GS	11.196 ± 0.055	465 ± 70	365 ± 77	11.20 ± 0.13	465 ± 60	365 ± 30
GS	10.11 ± 0.21	859 ± 33	2581 ± 88	10.11 ± 0.19	859 ± 94	2580 ± 250
GS	9.46 ± 0.16	955 ± 70	916 ± 65	9.46 ± 0.15	955 ± 95	921 ± 25
GS	8.434 ± 0.073	800 ± 74	181 ± 15	8.421 ± 0.065	801 ± 53	180 ± 19
ES	11.2 ± 1.7	300 ± 21	260 ± 21	10.4 ± 1.6	750 ± 210	1100 ± 260
ES	10.3 ± 1.9	590 ± 130	1194 ± 44	10.0 ± 1.9	598 ± 66	2150 ± 130
ES	9.9 ± 2.5	760 ± 29	2100 ± 130	9.6 ± 1.7	555 ± 73	1500 ± 320
ES	9.35 ± 0.72	800 ± 100	790 ± 51	8.9 ± 1.6	493.7 ± 9.1	332 ± 35
ES	9.04 ± 0.95	903 ± 36	89.9 ± 7.8	7.08 ± 0.82	495 ± 55	70.0 ± 4.3
ES	8.5 ± 1.4	895.4 ± 6.6	120.5 ± 6.2			
ES	8.05 ± 0.48	895 ± 50	155 ± 13			
ES	7.5 ± 1.3	809.6 ± 8.5	10.0 ± 1.5			
X				11.2 ± 1.9	301 ± 19	260 ± 13
X				10.3 ± 2.6	596 ± 60	1140 ± 220
X				9.9 ± 1.5	750 ± 53	1870 ± 140
X				9.35 ± 0.56	796 ± 61	862 ± 93
X				9.0 ± 1.1	905 ± 28	130 ± 12
X				8.6 ± 4.5	900 ± 73	82 ± 14
X				8.33 ± 0.81	802 ± 71	260 ± 19
X				7.33 ± 0.56	850 ± 41	20.0 ± 1.6
MS2	11.0 ± 1.8	810 ± 170	425 ± 78	11.00 ± 0.44	805 ± 31	425 ± 39
MS2	10.4 ± 2.1	550 ± 35	1110 ± 180	10.4 ± 1.4	550 ± 92	1390 ± 370
MS2	10.0 ± 2.4	710 ± 20	2950 ± 200	10.0 ± 1.9	708 ± 42	2610 ± 130
MS2	9.7 ± 1.6	734 ± 31	1200 ± 230	9.6 ± 1.4	734 ± 50	1280 ± 80
MS2	9.20 ± 0.58	663 ± 49	533 ± 42	9.2 ± 2.9	662 ± 43	500 ± 29
MS2	8.7 ± 3.3	705 ± 72	161 ± 11	8.7 ± 1.9	700 ± 110	170 ± 46
MS2	8.15 ± 0.53	960 ± 58	120 ± 10	8.35 ± 0.65	960 ± 100	170.1 ± 5.4
H_2_O	10.80 ± 0.23	533 ± 36	1300 ± 260	10.80 ± 0.23	533 ± 20	1300 ± 130
H_2_O	10.50 ± 0.25	721 ± 49	2430 ± 150	10.50 ± 0.25	721 ± 36	2430 ± 220
CC	10.6 ± 2.1	720 ± 180	−470 ± 230	10.6 ± 2.7	700 ± 170	−520 ± 120
CC	9.8 ± 3.6	301 ± 36	80.9 ± 3.9	10.0 ± 3.3	301 ± 34	52 ± 2
CC	9.4 ± 1.3	760 ± 160	67 ± 7	9 ± 2	760 ± 180	62.1 ± 2.6
CC	8.8 ± 1.2	881 ± 30	307 ± 50	8.84 ± 0.91	880 ± 100	316 ± 14
CC	8.15 ± 0.41	955 ± 70	190 ± 35	8.15 ± 0.28	955 ± 43	205 ± 25
CC	7.27 ± 0.59	913 ± 47	26.0 ± 1.5	7.3 ± 0.7	910 ± 130	21.7 ± 1.5
CC	5.95 ± 0.51	605 ± 71	7.00 ± 0.49	5.95 ± 0.55	605 ± 46	6.00 ± 0.51
CC	6.57 ± 0.56	900 ± 82	10.62 ± 0.61	6.57 ± 0.56	900 ± 60	10.92 ± 0.46

Before turning to a more detailed discussion about the existence of the X state in SNP, we would like to stress that the transfer time for the population of MS2 at ambient temperature is $k_{12}^{-1}=253\;\text{fs}$ for model 1, and $k_{1X}^{-1}+k_{X2}^{-1}=234\;\text{fs}$ for model 2, respectively. It is also found that the competing process ^1^ES → ^1^GS occurs on the same time scale—actually slightly faster—as the population of MS2. Hence, slightly less than 50% of the originally excited molecules switch towards the MS2 configuration, whereas the over-all switching ratio (i.e., with respect to the total number of molecules) is ∼10%. Both values are in good agreement with the data of Ref. 24, and regardless of the presence of an intermediate state.

The de-excitation of the MLCT state, considered in models 1 and 2, involves channels of different nature. Gallé *et al.* suggested that the ${\;}^{1}\text{ES}\to^{1}\text{GS}$ transition (model 1) leads to the population of a rather highly excited vibrational level of the GS state, whose thermalization then proceeds on a timescale of a few picoseconds.^36^ Such a channel is not considered in model 2. Instead, the de-excitation proceeds via population of the X state, which can be the same singlet excited state, ^1^ES, where the NO ligand is slightly rotated toward its side-on orientation, denoted as ^1^X in Fig. 1. On the one hand, the fast decay time (33 fs) of the ^1^ES signal, which is much faster than the transition to ^1^MS2 (*k*_12_, see Table I) or back relaxation to ^1^GS (*k*_10_), supports changes of the geometric configuration or even of the electronic state. A possible candidate for such a transient electronic state is the first excited triplet state, depicted as a green curve in Fig. 1. On the other hand, the timescale of this transition appears to be quite fast both for NO rotation and intersystem crossing (ISC), because of the large mass of the NO ligand and the smallness of the spin-orbit coupling. As a very rough estimate of the time needed for geometric reorganization, one can consider the 24 fs half-period of the 708 cm^−1^ ground state Fe–NO bending normal mode in the limit of small vibrations, which is closest to the NO rotation motion depicted in Fig. 1. In addition, a special comment needs to be added concerning the multiplicity of the X state. Although the potential energy surfaces of the first excited singlet and triplet states are almost parallel along the NO rotation coordinate, the analysis in the spirit of the vibronic coupling model suggests a crossing of singlet and triplet states along the totally symmetric stretching Fe–NO tuning mode with the ground state frequency of 818 cm^−1^ (Schaniel *et al.* reported a mode at 662 cm^−1^).^24^ This can make the ISC quite efficient and, thus, the photochemical pathway from ^1^ES to ^1^MS2 may be accompanied by an ultrafast double ISC (${\;}^{1}\text{ES}\to^{3}X\to^{1}\text{MS}2$) and represents a relaxation dynamics involving both NO rotation and Fe–NO stretching nuclear motions. However, the probability of such an ultrafast double ISC should be lower than that of the internal conversion (IC) ${\;}^{1}\text{ES}\to^{1}X\to^{1}\text{MS}2$ or ^1^GS. Note that unravelling the competition between multiple ISC and IC in transition-metal complexes can be a non-trivial task, as recent investigations show.^58^ For simplicity, one might consider that singlet and triplet MLCT states form a joint band and ^1^ES and ^3^ES as well as ^1^X and ^3^X signals should be treated together in the global fit analysis.

**FIG. 5.**
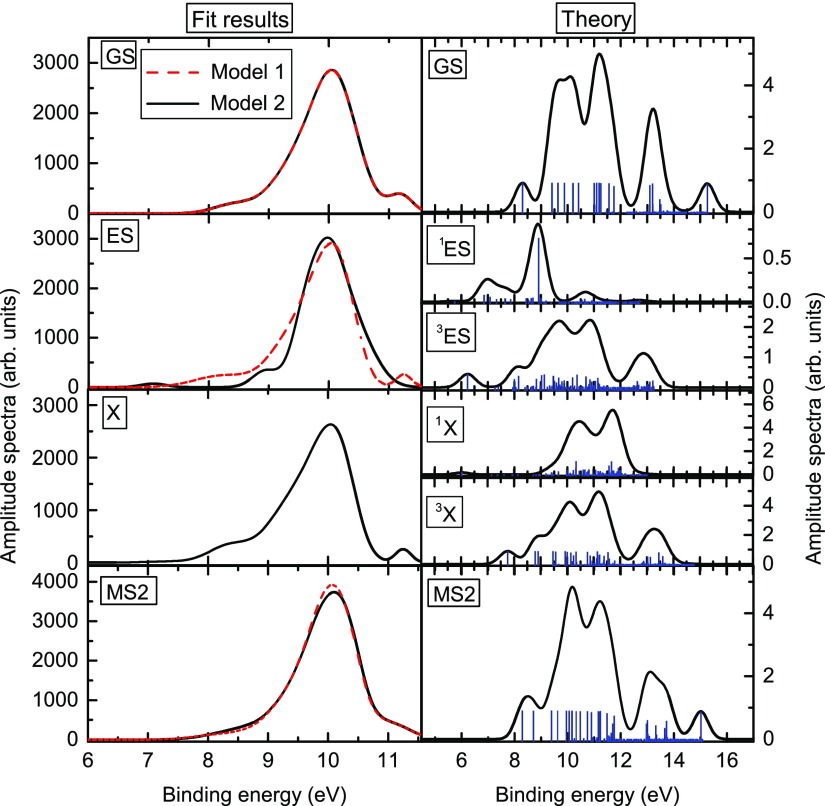
Comparison between PES amplitude spectra of SNP, as obtained from the global fits of models 1 and 2, respectively (left column). The spectra for cross-correlation and solvent are shown in the Appendix, Fig. 9. The right column shows XUV photoemission spectra calculated using TDDFT for the states GS, ${\;}^{1,3}\text{ES}{,}^{1,3}X$, and MS2. The solid curves have been obtained by convolution of the stick spectra (blue) with Gaussian functions of width *w* = 0.28 eV [see Eq. (A5)].

**FIG. 6.**
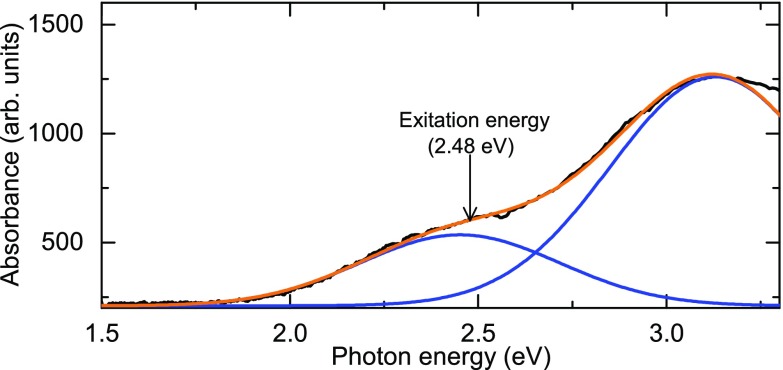
Absorbance spectrum of SNP in aqueous solution. The solid lines represent the decomposition of the measured spectrum into contributions of two different MLCT excitations. The excitation photon energy used in the PES experiment is indicated by the arrow.

**FIG. 7.**
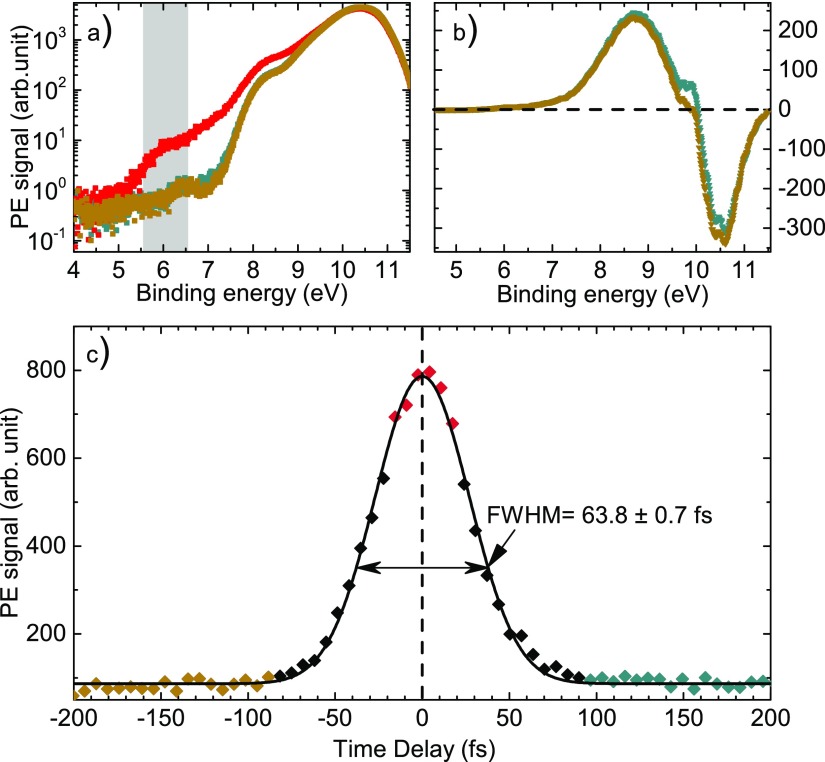
(a) Transient PE spectra of SNP, measured at time delays of −200 fs (brown), 0 fs (red), and +200 fs (blue). (b) Difference between the spectra obtained at time delay of zero and −200 fs (brown), and +200 fs (blue), respectively. (c) Transient signal in the time-domain, integrated between 5.55 eV and 6.55 eV [gray area in panel (a)]. The pump-probe cross-correlation trace is fitted by a Gaussian profile with an FHWM of (63.8 ± 0.7) fs, shown as black line.

**FIG. 8.**
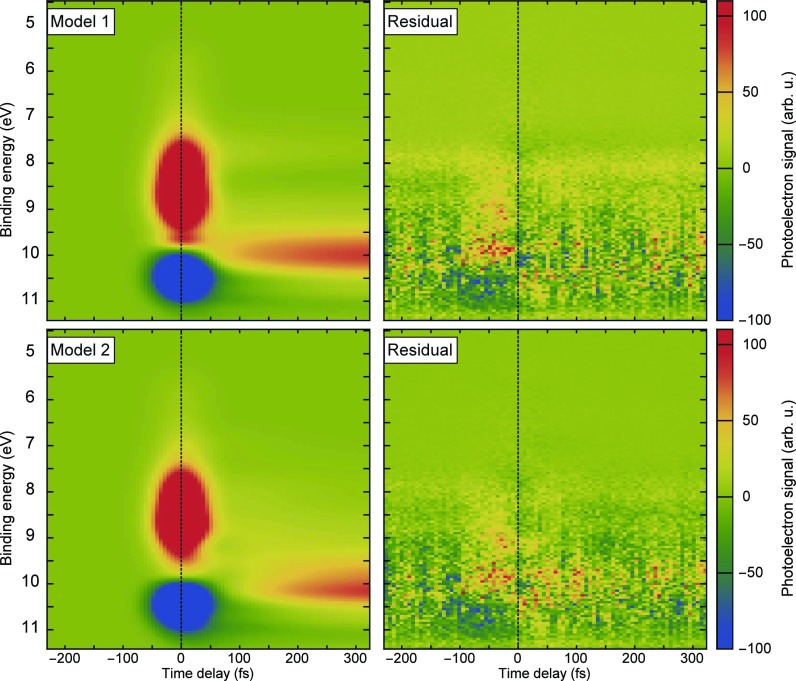
Global fit results and residuals for model 1 (upper panels) and model 2 (lower panels), respectively.

Unfortunately, the PES spectra originating from different electronic states at different geometric configurations are quite similar to each other with an exclusion being the spectrum of ^1^ES, see Fig. 5. This fact, which is probably intimately connected to the weakness of the NO^+^ photoionization yield in the investigated PES energy region, does not allow us to unambiguously assign the transient signal to the triplet electronic state. Therefore, further experiments are needed to clarify the multiplicity of the X state. Nevertheless, the calculated potential energy curves and the fast decay of the unique transient signal assigned to ^1^ES strongly suggest involvement of the intermediate state ^1,3^X, either singlet or triplet.

Finally, we would like to note that besides the ultrafast transition ${\;}^{1}\text{ES}\to^{1}X$, Lynch *et al.* suggested a parallel ultrafast (10 fs) back relaxation ${\;}^{1}\text{ES}\to^{1}\text{GS}$, which was assigned to a stimulated emission process.^32^ From our data analysis, we cannot find evidence for such a transition. In fact, both models 1 and 2 yield time constants of ∼200 fs for the back transition toward GS. Further, to the best of our knowledge, there have been no reports so far on the luminescent properties of SNP, let alone stimulated emission. This indicates that any relaxation processes are of non-radiative nature. As considered by Gallé *et al.*, the transition ${\;}^{1}\text{ES}\to^{1}\text{GS}$ results in a highly excited vibrational state of GS, whose thermalization proceeds on a timescale of a few picoseconds.^36^ Based on these findings, we consider that a back relaxation ${\;}^{1}\text{ES}\to^{1}\text{GS}$ on a 10-fs timescale is very unlikely, leaving either the scenario of Refs. 36 and 24 (model 1), or the indirect path of model 2, as discussed in this work.

## V. CONCLUSION AND OUTLOOK

In conclusion, we have shown that time-resolved photoelectron spectroscopy is well suited for investigations of linkage isomers, using Na_2_[Fe(CN)_5_NO] as a model complex. The temporal resolution of the experiment is sufficient to observe ultrafast photoexcitation and subsequent relaxation processes, resulting in the population of metastable state MS2 in less than 240 fs. Our investigations thus corroborate the results of previous reports concerning the excitation and relaxation kinetics.^17,24,32,36^

With the aid of TDDFT calculations, we could further identify the absolute binding energies of the involved electronic ground and excited states, and reveal the presence of a short-lived intermediate state in the relaxation pathway to the metastable isomerized state MS2. However, further investigations are needed to identify the multiplicity of this state.

Based on these findings, investigations of the early-time kinetics of novel linkage isomers by means of transient PES become feasible. Owing to our recent progress in experimental technique, utilization of ionic-liquid droplets instead of liquid jets will facilitate such investigations even for non-abundant complexes, which are available only on the milligram scale.^59^

## Figures and Tables

**FIG. 9.**
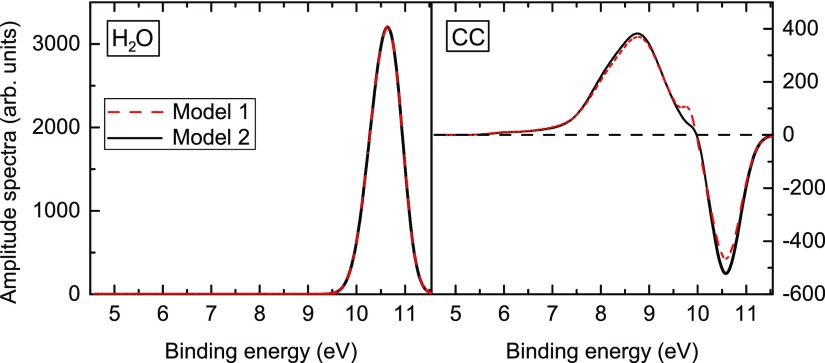
Amplitude spectra obtained from global fits of models 1 and 2 for the solvent (H_2_O) and cross-correlation (CC) signals. The corresponding cuts and 2D-plots are shown in Figs. 3 and 8, respectively.

## APPENDIX: DATA ANALYSIS ROUTINE

### 1. Data treatment

A series of scans over the time delay range between −230 fs and +330 fs was accumulated in the experiment. While combining the series, the data sets were corrected for the delay-dependent energy shift and the drift of the zero time delay. The energy shift was caused by the space-charge effect induced by the pump beam in the liquid sample. This shift was shown to be dependent on the time delay between the pump and probe pulses.^60^ Therefore, each spectrum of a given series needed to be corrected separately. The ionization signal from the Fe 3d(2b_2_,6e) orbital, giving rise to a well distinguished energy peak in each XUV spectrum, was used as a reference. A Gaussian fit was used to determine the central kinetic energy of this peak and the binding energy was calculated as the difference between the XUV photon energy (32.55 eV) and the kinetic energy. The drift of the zero time delay was caused by changes in the environmental conditions in the lab and could reach a value of 120 fs during the day. This drift was corrected according to the center position of the cross-correlation signal in each time-delay scan.

### 2. Determination of temporal resolution

Figure 7(a) shows transient spectra at different delay times. While the signal at Δ*t* = −200 fs exhibits the spectrum of GS, the one at Δ*t* = 0 fs is clearly dominated by the cross-correlation (CC) signal. At positive delay, Δ*t* = +200 fs, the original signal is recovered to a large extent [cf. Fig. 7(b)]; the subtle differences observable in Fig. 7(a), e.g., around 10 eV, indicate the transient states ES, X, and MS2. From the time-dependency of the signal, integrated between 5.55 eV and 6.55 eV [gray area in Fig. 7(a)], we deduce the duration of the CC to be (63.8 ± 0.7) fs (FWHM), as illustrated in Fig. 7(c).

### 3. Details of the fitting routine

The experimental data set was analyzed using a global-fit approach. We assume either of the two kinetic models 1 or 2, as explained in the main text. Model 1 includes three states (GS, ES, and MS2), while model 2 assumes an additional intermediate state X. The corresponding rate constants are denoted by *k_if_* [see Figs. 4(a) and 4(b)]. The pump intensity is assumed to have a Gaussian temporal envelope with unit amplitude; the actual intensity measure is contained in the fit parameter *a*, describing the time-dependent pump rate

$$I_{\text{pump}}(t)=\text{exp}\;\left[\frac{-{\left(t-t_{0}\right)}^{2}}{2\sigma_{\text{pump}}^{2}}\right].$$ (A1)

The respective rate equation system for model 1 reads

$$\begin{array}{c} \frac{d[\text{GS}]}{dt}=-a\;I_{\text{pump}}(t)[\text{GS}]+k_{10}[\text{ES}]+k_{20}[\text{MS}2],\\ \frac{d[\text{ES}]}{dt}=+a\;I_{\text{pump}}(t)[\text{GS}]-k_{10}[\text{ES}]-k_{12}[\text{ES}],\\ \frac{d[\text{MS}2]}{dt}=+k_{12}[\text{ES}]-k_{20}[\text{MS}2]. \end{array}$$ (A2)

For model 2, we have

$$\begin{array}{c} \frac{d[\text{GS}]}{dt}=-a\;I_{\text{pump}}(t)[\text{GS}]+k_{X0}[\text{ES}]+k_{20}[\text{MS}2],\\ \frac{d[\text{ES}]}{dt}=+a\;I_{\text{pump}}(t)[\text{GS}]-k_{1X}[\text{ES}],\\ \frac{d[X]}{dt}=+k_{1X}[\text{ES}]-k_{X0}[X]-k_{X2}[X],\\ \frac{d[\text{MS}2]}{dt}=+k_{X2}[X]-k_{20}[\text{MS}2]. \end{array}$$ (A3)

In both cases, the initial condition at *t* → −*∞* is [GS] = 1, while all other states are unpopulated. Solving the kinetic equations (A2) or (A3) results in a population-density matrix, wherein the rows contain the normalized time-dependent population densities of the real states of SNP. In order to account for the contribution of the solvent signal, we assume a constant population density of unity. The time-dependent CC signal is represented by the amplitude-normalized Gaussian Eq. (A1), i.e., identical to the pump intensity. The latter two signals are appended as additional rows to the population-density matrix. To obtain the *actually observable* kinetic matrix *T*(*t*), including the water and CC state, the rows of the population-density matrix are convolved with the area-normalized Gaussian probe-pulse intensity

$$I_{\text{probe}}(t)=\frac{1}{\sqrt{2\pi }\sigma_{\text{probe}}}\text{exp}\;\left[\frac{-{\left(t-t_{0}\right)}^{2}}{2\sigma_{\text{probe}}^{2}}\right].$$ (A4)

For all the states thus obtained, the corresponding PES spectra are represented as columns of the energy-dependent amplitude matrix *A*(*E*). While the photoemission spectra of the solvent and SNP states are strictly positive, the CC signal may assume also negative values, where negative amplitudes account for the bleaching arising from the depletion of GS and solvent states by the pump pulse. In this way, bleaching does not imply negative population densities of the SNP states. Here, the amplitude spectra for each state *i* are defined as

$$A_{i}(E)=\sum_{j=1}^{n_{i}}A_{ij}\;\text{exp}\;\left[-4\;\ln\;(2)\frac{{\left(E-E_{ij}\right)}^{2}}{w_{ij}^{2}}\right].$$ (A5)

Equation (A5) represents a decomposition of the photoemission spectrum of state *i* into a sum of *n_i_* photoemission bands described by Gaussian profiles. Finally, the time- and energy-dependent signal matrix *D*_mod_ is obtained by a matrix multiplication

$$D_{\text{mod}}=A(E)\;T(t).$$ (A6)

The fit is performed by minimizing the objective function

$$\chi^{2}={\left|\left|\frac{D_{\text{exp}}-D_{\text{mod}}}{\kappa }\right|\right|}^{2},$$ (A7)

where *κ* denotes the standard deviation of the respective data points. According to the measurement process of the TOF spectrometer, we assume the standard deviation to be the square root of the data value, i.e., $\kappa_{ij}=\sqrt{D_{\text{exp},ij}}$. Error estimation is performed by numerically calculating the Hessian of the objective function.

The best fit parameters, including errors, are shown in Tables I and II, respectively. The residuals are shown in Fig. 8.
